# Iron overload disrupts bone homeostasis via TfR1-dependent ferroptosis and cGAS/STING-driven pyroptosis in pyogenic spondylitis

**DOI:** 10.3389/fimmu.2026.1760434

**Published:** 2026-06-30

**Authors:** Wenchao Xu, Qinpeng Xu, Hongdong Tan, Xiaodong Liu, Jiaju Ma, Fei Jia, Heng Yang, Meimei Zheng, Jianlong Li, Xingang Cui, Xingzhi Jing, Xiaoyang Liu

**Affiliations:** 1 Department of Spine Surgery, Shandong Provincial Hospital Affiliated to Shandong First Medical University, Jinan, China; 2 Shandong Public Health Clinical Center, Shandong University, Jinan, Shandong, China; 3 Department of Spine Surgery, Shandong Provincial Hospital, Shandong University, Jinan, China; 4 Department of Neurology, The First Affiliated Hospital of Shandong First Medical University & Shandong Provincial Qianfoshan Hospital, Jinan, China

**Keywords:** bone loss, cGAS/STING pathway, iron overload, osteoblast, osteoclast, oxidate stress, pyogenic spondylitis, TFR1

## Abstract

**Introduction:**

Pyogenic spondylitis (PS) accompanies with diverse destruction, especially the subsequent bone destruction, which leads to spine instability and severe neurological disability. However, the mechanism underlying bone loss induced by infection has not been elucidated. In this study, we aimed to reveal a novel mechanism of bone destruction in PS.

**Methods:**

To certify the involvement of iron overload in PS-induced bone loss, vertebrae samples were collected and evaluated from patients with PS. Next *Staphylococcus aureus* (*S. aureus*, ATCC 25923) was used to induce bone infection *in vivo* and *in vitro*, and relevant markers were investigated. Then, experiments using siRNA targeting transferrin receptor-1 (TfR1), an iron chelator (DFO), and the TfR1 inhibitor Ferristatin II were conducted to investigate the role of TfR1-induced iron overload and ferroptosis in PS-induced bone destruction.

**Results:**

Infected vertebral specimens from PS patients showed iron overload and increased TfR1 expression, which was also observed in *S. aureus* -infected MC3T3-E1 cells. Excessive iron leads to osteoblast ferroptosis and osteogenic activity via iron overload and oxidative stress injury, which was inhibited by TfR1 siRNA or DFO. Meanwhile, iron overload promoted mtDNA leakage and activated the cGAS/STING pathway, contributing to NLRP3-associated pyroptosis and impaired osteogenesis. In addition, *S. aureus* -induced iron overload in osteoclasts promoted osteoclastogenesis, which was also ameliorated by TfR1 siRNA or DFO. *In vivo*, Ferristatin II reduced iron deposition, suppressed TfR1 expression, and preserved trabecular architecture in PS rats.

**Conclusions:**

Our research indicates that *S. aureus* infection triggers iron overload in infected bone tissue via the promotion of TfR1 expression, finally contributing to osteoblast ferroptosis and bone destruction. Targeting TfR1-mediated iron influx and ferroptosis is a novel therapeutic strategy for the treatment of bone loss induced by PS.

## 1 Introduction

Pyogenic spondylitis (PS) refers to infections involving spinal bony structures, intervertebral discs, epidural spaces, and adjacent myofascial tissues. Its incidence rate is gradually rising due to population aging and the inappropriate use of antibiotics (1). The pathogenic bacteria of PS are diverse, the most common of which is *Staphylococcus aureus(S. aureus)*, accounting for approximately 40% of all PS cases (2). It is difficult for antibiotics to reach infected tissues because of poor blood circulation and bacterial biofilm formation. Local bacteria proliferate rapidly, release invasive enzymes, induce inflammatory cell infiltration, and disturb bone homeostasis imbalance, which subsequently contributes to bone destruction, spinal instability, neurological disabilities, and even death (3). In recent decades, researchers have been exploring the mechanisms of bone loss in PS. Unfortunately, current studies have not clearly illustrated the pathogenesis of bone loss in PS, which greatly hinders clinical therapeutic progress and impairs patients’ quality of life (4). Thus, revealing a novel pathogenesis of bone loss is significant for developing new therapies.

Ferroptosis is a novel form of regulated cell death triggered by iron accumulation and lipid peroxidation (5), leading to mitochondrial abnormalities characterized by swelling, increased membrane density, decreased or absent cristae, and rupture of the outer membrane (6). Ferroptosis is strongly associated with the inflammatory response. Both lipid peroxidation and dysfunctional redox processes trigger the activation of inflammatory cells and pathways, while pro-inflammatory factors can exacerbate lipid peroxidation and oxidative stress processes (7). A recent study indicates that ferroptosis‐related genes may serve as potential biomarkers for *S. aureus*‐induced osteomyelitis (8). Bacterial infection is closely related to ferroptosis in host cells. Researches show that *S. aureus* can adapt lysosomes and autophagosomes to new conditions, enabling them to compete with iron carriers to interact with ferroptosis regulators (9, 10). Inhibiting ferroptosis can alleviate inflammation in endometritis and mastitis caused by *S. aureus* (11, 12). Our previous studies revealed that bone loss and destruction may originate from inflammatory infiltration (13, 14). It is critical to confirm whether iron overload and ferroptosis play significant roles in *S. aureus*‐induced pyogenic spondylitis. It’s significant to clarify whether ferroptosis involves bone loss in PS, aiming to improve patients’ quality of life.

Cellular iron balance is mainly regulated by the influx of iron ions. In the bloodstream, Fe^3+^ binds to transferrin (Tf) to form a holo-transferrin complex. This complex then specifically binds to transferrin receptor-1 (TfR1) on the cell surface, facilitating its cellular uptake via receptor-mediated endocytosis (15). TfR1 is a transmembrane glycoprotein that is widely expressed in various tissues and organs and plays a critical role in mediating cellular iron influx and maintaining intracellular iron homeostasis (16). In certain central nervous system diseases, the expression of TfR1 is significantly higher than that in normal tissues. This overexpression of TfR1 can increase intracellular iron concentrations, resulting in disrupted iron homeostasis, making TfR1 a valuable target for treating diseases (17, 18).

In the present study, we observed significant iron accumulation in patients with pyogenic spondylitis. To further elucidate the role of ferroptosis, we established an infection model to explore whether iron overload is involved in bone destruction in PS. Finally, MC3T3-E1 cells and osteoclasts were cultured to investigate the effects of iron overload and ferroptosis mediated by oxidative stress. Our study aimed to clarify a novel pathogenesis of bone loss in PS and subsequently provide new strategies to prevent and treat the devastating effects of PS.

## 2 Materials and methods

### 2.1 Reagents

Desferoxamine (DFO, catalog #D9533), L-Ascorbic Acid (catalog #A5960), Ferric ammonium citrate (FAC, catalog #F5879), Ferristain I (Fer-I, catalog #SML0583) and β-Glycerophosphate (catalog #9422) were purchased from Sigma-Aldrich (St. Louis, MO, USA). N-acetyl-L-cysteine (NAC, catalog #S1623) was purchased from Selleck (Houston, USA). MedChenExpress (China) supplied M-CSF (catalog #HY-P7085), Dexamethasone (catalog #HY-14648) and Ethidium bromide (EtBr, catalog #HY-D0021). R&D Systems (USA) was the source of acquisition for Rankl (catalog #462-TEC-010/CF). Ferristain II (Fer-II, catalog #NO.36621) was purchased from Cayman Chemical Co. (Ann Arbor, Michigan, USA). Tartrate-Resistant Acid Phosphatase Stain Kit (catalog #G1492) was purchased from Solarbio (China).

### 2.2 Clinical study

This study was approved by the Ethics Review Committee of Shandong Provincial Hospital affiliated to Shandong First Medical University (No. SDNSFC 2023-0198). All participants were adults and completed a written informed consent form. Our study is in accordance with the principles of the Declaration of Helsinki.

In this study, we collected bone tissue and serum samples from 10 non-infected patients and 12 patients with *S. aureus*-induced PS confirmed by culture of tissue or blood. To minimize confounding factors, patients with autoimmune diseases (e.g. rheumatoid arthritis) or ankylosing spondylitis were excluded from this study. All specimens were decalcified in 10% EDTA for at least 4 weeks, and then stained for histological and immunohistochemical analysis.

### 2.3 Cell line and *S. aureus* infection *in vitro*

Mouse calvaria-derived osteo-precursor cells MC3T3-E1, acquired from Procell Life Science & Technology Co. Ltd. (catalog #CL-0378, China), were cultured under 5% CO2 at 37 °C in α-minimum essential medium (α-MEM, catalog #12571063, Gibco, USA) with supplementation of 10% fetal bovine serum (FBS, catalog #A5670701, Gibco, USA), 1% penicillin/streptomycin (catalog #C0222, Beyotime, China). Every 72h, the medium was replaced. After reaching the desired confluency, the cells were collected using trypsin-EDTA (catalog #25200114, Gibco, USA) and then passaged. Cells from the 10th to 12th generation were used for subsequent experiments. *Staphylococcus aureus* (*S. aureus*, ATCC 25923) was incubated in trypticase soy agar (TSA) medium at 37 °C for 18h. After 18h, *S. aureus* was harvested and resuspended in sterile PBS at a concentration of 1×10^9^ CFU/ml according to the optical density measurements. The suspension was then diluted to a bacterial concentration of 1×10^7^ CFU/ml.

To mimic iron overload, FAC and DFO were implemented as previously described (19). Briefly, MC3T3-E1 cells were pretreated with 100μM FAC for 72 h, followed by continuous treatment with 10μM DFO for an additional 24 h. In separate experiments, MC3T3-E1 cells were first treated with 10μM DFO for 24h, 20μM TfR1 siRNA for 72h, 10μM Fer-I for 48h, 20μM STING siRNA for 72h, 1mM NAC for 24h, and subsequently infected with S. aureus at a multiplicity of infection (MOI) of 50:1.

After 4h of incubation with *S. aureus*, MC3T3-E1 cells were washed with PBS, treated with 20mg/mL lysostaphin for 30 min to kill bacteria extracellularly, and cultured in fresh medium.

### 2.4 Animal selection and grouping

Twenty-four female Wistar rats aged 8 weeks old were randomly divided into four groups (n = 6 per group): control, *S. aureus*, *S. aureus* + Fer-II and Fer-II groups. The animals were housed in individual cages for at least seven days before surgery and had free access to food and water. Infectious model was created by injecting 2×10^5^/20ul of *S. aureus* suspension into the vertebra of rats (20). The control group was injected with 20ul PBS. Rats in the Fer-II and *S. aureus* + Fer-II group were intraperitoneally injected with Fer-II (10 mg/kg/d) according to a previous study (21). All animals successfully completed the experiment and euthanized with carbon dioxide 2 weeks later. The involved vertebrae were collected for micro-computed tomography (CT), histological, and immunohistochemical examinations. All animal procedures were approved by the Animal Care Committee of Shandong Provincial Hospital affiliated to Shandong First Medical University (No. SDNSFC 2023-0198).

### 2.5 Micro-computed tomography analysis

The collected vertebral samples were fixed in 4% paraformaldehyde for 24h, followed by careful removal of the surrounding skin, muscles, and ligaments. Micro-CT scanned the infected with a 11.6μm resolution, 70 kVp and 57μA (ScancoViva-CT80 system, Switzerland). The 3D reconstruction software (ScancoViva-CT80 system, Switzerland) was used to process the primary data and to produce the bone microstructure report, including trabecular number (Tb.N), trabecular thickness (Tb.Th), trabecular separation (Tb.Sp), and bone volume fraction (BV/TV).

### 2.6 Histological staining and immunohistochemistry analysis

After decalcification, samples were embedded, sectioned at a 5μm thickness and evaluated by Hematoxylin-eosin (HE) staining, tartrate-resistant acid phosphatase (TRAP) staining, and Dab-enhanced Prussian blue staining to present trabecular bone destruction, osteoclast expression, and iron content. Briefly in immunohistochemistry, the sections were deparaffinized, rehydrated, and blocked with 5% BSA for a duration of 30 min at a temperature of 37°C. The Primary antibodies (TfR1 1:500, Runx-2 1:1000, GPX4 1:200 obtained from Abcam) were incubated at 4°C overnight. Slides were then treated with secondary antibodies, imaged on an Olympus light microscope (Thermo Fisher Scientific, IX53), and analyzed to count positive cells using ImageJ version 2.90.

### 2.7 Cell viability assay

MC3T3-E1 cells were seeded into 96-well culture plates (10000 cells/well) and then infected with *S. aureus* for 4h at MOI of 0, 1:1, 10:1, 50:1, and 100:1. The extracellular bacteria were killed with 20 mg/mL lysostaphin for 30 min. Then, 100μl of fresh medium and 10μl of CCK-8 solution (catalog #C0038, Beyotime, China) were added in to each well and incubated at 37°C for 1h. The absorbance at 450 nm was measured on a microplate reader.

### 2.8 Malondialdehyde measurement

MC3T3-E1 cells were seeded into 12-well culture plates (2×10^5^ cells/well) and intervened according to previously described methods. Cellular proteins (0.1mL) were mixed with 0.2ml MDA detection working solution (catalog #S0131S, Beyotime, China). After cooling the mixture, 200μl of supernatant was added to a 96-well plate, and the absorbance was measured at 532 nm to quantify MDA levels using a microplate reader.

### 2.9 C11-BODIPY staining

Briefly, C11-BODIPY staining was performed according to the manufacturer’s instructions (C11-BODIPY catalog #S0043S, Beyotime, China). Intracellular lipid peroxides were determined under a fluorescence microscope (Axio Observer 3; Carl Zeiss).

### 2.10 Alkaline phosphatase activity assay

ALP expression was used to assess the early osteogenic differentiation of MC3T3-E1 cells (22). Typically, ALP activity exhibited its maximum level following a 7-day period of osteogenic culture induction. Briefly, the MC3T3-E1 cells were cultured in 12-well plates (2×10^5^ cells/well). On the seventh day of osteogenic culture, the cells were stained using an ALP kit (catalog #P0321S; Beyotime, China) according to the manufacturer’s instructions. The mixture of cell lysates and buffer was incubated at 37°C for 20 min and determined using a bicinchoninic acid (BCA) protein assay kit (catalog #AR0146, Boster, China). ALP activity was calculated as the OD value at 405 nm per milligram of total protein.

### 2.11 Staining with alizarin red

On the 21st day of osteogenic culture, cells were fixed with 4% paraformaldehyde and then treated with alizarin red solution (catalog #G1038, Servicebio, China) for 30 min at room temperature. After washing with 10% cetylpyridinium chloride (catalog #6004-24-6, Sigma-Aldrich, USA) for 1h, the mineralized nodules were quantified via spectrophotometric absorbance measurements of optical density at 570 nm.

### 2.12 Annexin V-FITC/PI staining and flow cytometry

MC3T3-E1 cells were stained with Annexin V-FITC/PI for 20 min in the dark at room temperature (catalog #MA0220; Meilunbio, China). The apoptosis rate was calculated as the total number of apoptotic cells (sum of early apoptosis (annexin V+/PI−) and late apoptosis (annexin V+/PI+)) divided by the total cell count.

### 2.13 Reactive oxygen species assay

The levels of ROS in MC3T3-E1 cells were evaluated according to the manufacturer’s instructions (catalog #S0033, Beyotime, China). Cells were washed with PBS and incubated with 10μM DCFH-DA fluorescent probe in the dark for 20 min at 37°C. After incubation, the cells were rinsed twice with serum-free α-MEM and measured under a fluorescence microscope (Axio Observer 3; Carl Zeiss). To quantify intracellular ROS levels, cells were collected and resuspended in serum-free α-MEM and then evaluated with a FACSCalibur flow cytometer (BD LSRFortessa) to determine the mean fluorescence.

### 2.14 Determination of mitochondrial membrane potential

Mitochondrial membrane potential (MMP) was measured using a catalog C2006 kit (Beyotime, China). MC3T3-E1 cells were exposed to JC-1 staining working solution or an equivalent amount of medium without serum for 20 min at 37°C in the dark. After washing twice, the cells were examined using a fluorescence microscope (Axio Observer 3; Carl Zeiss).

### 2.15 siRNA transfection

Specific siRNA transfection procedures were conducted to knockdown the expression of TfR1 in MC3T3-E1 cells and bone marrow macrophages (BMMs) using a RiboFECTTMCP kit (catalog #C10511-05, Ribo Bio, China). The cells were transiently transfected with 20uM siRNA for 72h following the manufacturer’s instructions. The sequences of mouse siRNA were as follows: #1 siTfR1:5′-CAGAAAAGCUAUUUGGAAATT-3,’ #2 siTfR1:5′-GGAUAUGGGUCUAAGUCUATT-3,’ and #3 siTfR1:5′-GGAU-UUAGACCCAGCAGAATT-3.’ #1 siSTING:5′-GGUCAUACUACAUUGGGUATT-3,’ #2 siSTING:5′-GCACAUUCGUCAGGAAGAATT-3,’ and #3 siSTING:5′-GGCAAAGGAUCCACCAAAUTT-3.’ After transfection, the knockdown efficiency was confirmed by western blotting, and the most effective TfR1 siRNA was selected for subsequent analysis.

### 2.16 Western blotting analysis

The cells were lysed on ice for 60 min using RIPA lysis buffer containing proteinase and phosphatase inhibitor cocktails from CWBIO (China). After quantification of protein concentration (Beyotime, China), samples were separated on 10% SDS- PAGE gels and were subsequently transferred onto PVDF membranes (catalog #ISEQ00010, Millipore, USA) by electroblotting. The membranes were blocked in 5% non-fat dry milk for 2h at room temperature and incubated with the primary antibodies overnight at 4°C and then with peroxidase-conjugated secondary antibodies for 2h. Images were obtained using a BandScan scanner (Bio-Rad, USA), quantified using ImageJ version 2.90 and normalized to GAPDH or β-actin expression. The following primary antibodies were used: TfR1 (catalog #ab214039, Abcam, 1:1000), NCOA4 (catalog #ab314553, Abcam, 1:1000), SLC7A11 (catalog #26864-1-AP, Proteintech, 1:1000), GPX4 (catalog #ab125066, Abcam, 1:1000), FTH1 (catalog #BM4487, Boster, 1:1000), FTL (catalog #10727-1-AP, Proteintech, 1:1000), Bcl-2 (catalog #26593-1-AP, Proteintech, 1:1000), BAX (catalog #60267-1-Ig, Proteintech, 1:5000), NLRP3 (catalog #ab270449, Abcam, 1:1000), Caspase-1 (catalog #F0461, Selleck, 1:1000), il-1β (catalog #ab315084, Abcam, 1:1000), ASC (catalog #10500–1-AP, Proteintech, 1:1000), Runx-2 (catalog #ab192256, Abcam, 1:1000), NFAT2 (catalog #ab25916, Abcam, 1:1000), CTSK (catalog #ab187647, Abcam, 1:1000), cGAS (catalog #ab252416, Abcam, 1:1000), STING (catalog #F1N19, Selleck, 1:1000), GAPDH (catalog #10494-1-AP, Proteintech, 1:5000), β-actin (catalog #10494-1-AP, Proteintech, 1:10000).

### 2.17 Ferrous iron detection

MC3T3-E1 cells were stained with 1μM FerroOrange (catalog #F374, Dojindo, Japan) in HBSS for 45 min at 37 °C in the dark. Subsequently, cells were washed three times with HBSS and imaged using a fluorescence microscope (Axio Observer 3; Carl Zeiss).

### 2.18 Immunofluorescence staining

After treatment, cells were fixed with 4% paraformaldehyde for 15 min, permeabilized using 0.5% Triton X-100 incubated for 20 min at room temperature. After blocking with 5% BSA for 2h at room temperature, cells were then incubated with NLRP3 (catalog #ab270449, Abcam, 1:200) antibodies overnight at 4°C and then with the FITC-labeled secondary antibody against rabbit (A0562, Beyotime, China 1:500) for 1.5h at 37°C. Finally, cells were treated with 4,6-diamidino-2-phenylindole (DAPI) for 10 min.

### 2.19 Osteoclast culture and *S. aureus* infection

To generate osteoclasts (OCs) from enriched BMMs, bone marrow was harvested from the long bones of 6-week male mice, and cells were cultured in α-MEM plus 10%FBS and 50ng/ml of M-CSF for 3 days and then differential cultured for 5 days with α-MEM medium containing 10%FBS, 50ng/ml of M-CSF and 50ng/ml RANKL. To induce intracellular iron overload, BMMs were incubated with 200μM FAC for 16 h (23).

To mimic infection, BMMs were treated with *S. aureus* for 30 min at an MOI of 1:1 at 37 °C according to the previous report (24) and then sterilized by 20mg/mL lysostaphin.

### 2.20 Tartrate-resistant acid phosphatase assay

TRAP staining was performed as previously described (25). Briefly, cells were washed with PBS twice, fixed with 4% paraformaldehyde for 10 min, and then stained with TRAP staining solution for 1h at 37 °C according to the protocol. Multinucleated TRAP-positive cells were considered as osteoclasts.

### 2.21 mtDNA depletion

To induce mtDNA depletion in MC3T3-E1 cells, cells were cultured in α-MEM containing 10% FBS, with an addition of 100 ng/mL ethidium bromide (EtBr) for 72h.

### 2.22 Statistical analysis

Multiple comparisons were performed using one-way analysis of variance (ANOVA) followed by Tukey’s test for comparisons between multiple groups, such as WB and immunohistochemistry analyses. For Western blotting data expressed as relative fold change, Student’s t-test or one-way ANOVA with Dunnett’s test was used for pairwise comparisons and multi-group comparisons, respectively. Results are represented as mean ± SD, and statistical significance was set at p < 0.05. All analyses were performed using the GraphPad Prism software (version 10.5).

## 3 Results

### 3.1 Bone loss and imbalanced iron homeostasis in pyogenic spondylitis

**Figure 1:**
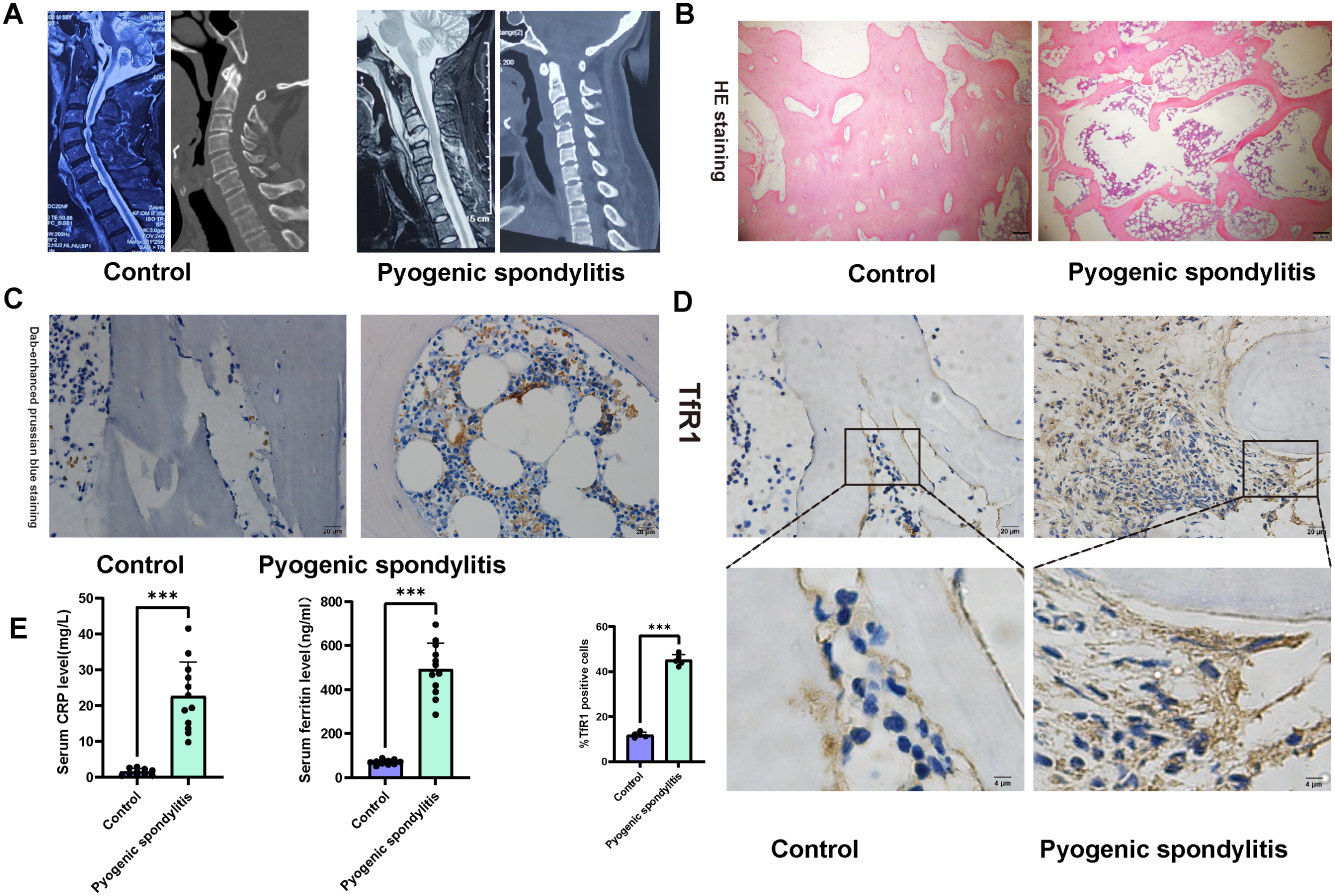
Pyogenic spondylitis is characterized by bone destruction and iron deposition. **(A)** Representative CT images showing marked bone destruction, and MRI demonstrating inflammatory edema and paravertebral abscess formation. **(B)** HE staining of vertebral specimens from non-infected and infected groups (scale bar = 200μm). **(C)** Dab-enhanced Prussian blue staining showing iron deposition in non-infected and infected specimens (scale bar = 20μm). **(D)** Representative immunohistochemical staining of TfR1 in vertebral tissues from non-infected and infected groups (scale bar = 20 μm), the lower panels show magnified views of the boxed regions(Scale bar = 4μm). Data are presented as mean ± SD, n = 5. ***p < 0.001. **(E)** Serum levels of C-reactive protein (CRP) and ferritin in the control (n = 10) and infected groups (n = 12). Data are presented as mean ± SD. ***p < 0.001.

First, we identified bone loss induced by infection. CT images revealed obvious bone loss characterized by bone destruction in PS patients, as indicated by MR images, whereas the control group showed no bone loss (**Figure 1A**). HE staining further confirmed local infection, characterized by obvious inflammatory cell infiltration and severe bone loss, especially a decrease in bone trabeculae (**Figure 1B**). PS patients in the infection group exhibited higher CRP level and ferritin level (Figure E). Dab-enhanced Prussian blue staining revealed increased Fe^3+^ content in the PS group compared to the control group (**Figure 1C**). Furthermore, the iron metabolism-related TfR1 was upregulated by infection (**Figure 1D**). These findings suggest that bone loss in patients with PS may be related to dysfunctional iron homeostasis.

### 3.2 *S. aureus* promoted MC3T3-E1 cells ferroptosis and inhibited its osteogenic differentiation

**Figure 2:**
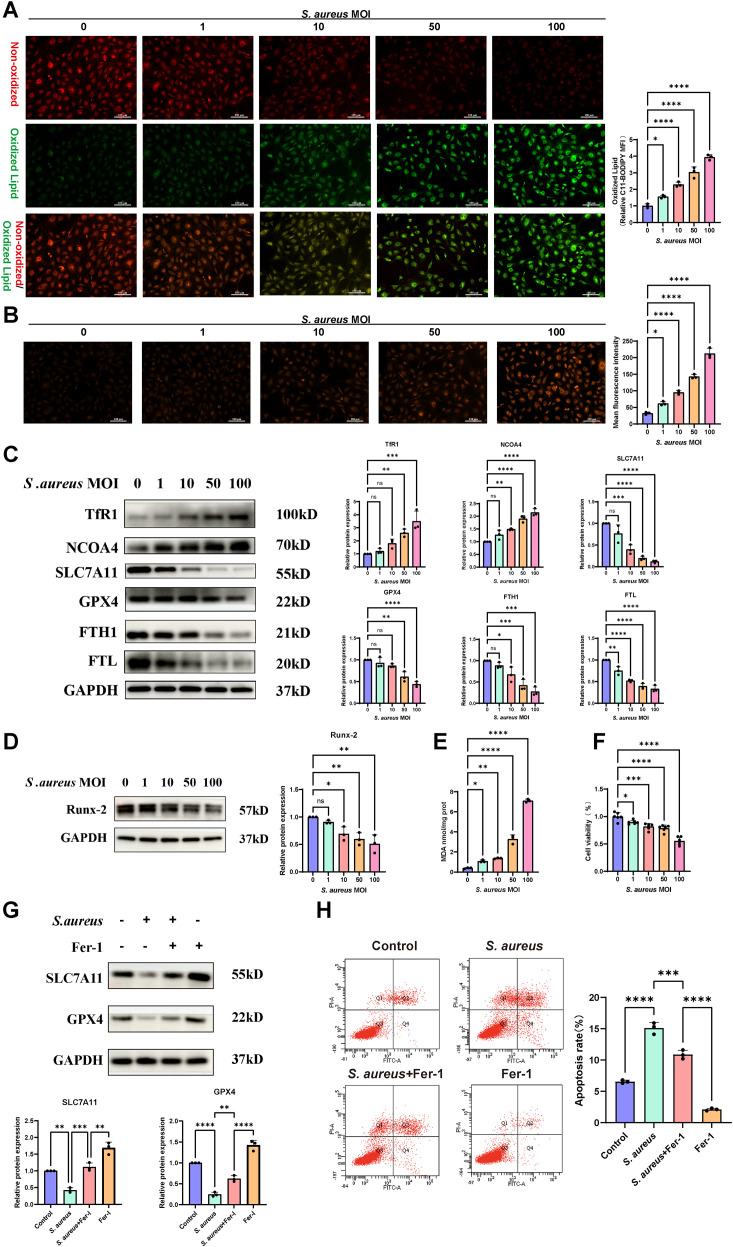
*S. aureus* infection promotes iron influx and osteoblast ferroptosis *in vitro*. **(A)** Representative fluorescence images of C11-BODIPY staining indicating lipid peroxidation levels (scale bar = 100μm). **(B)** Intracellular Fe²^+^ levels in MC3T3-E1 cells following *S. aureus* infection at different MOI (scale bar = 100μm). **(C)** Protein expression of TfR1, NCOA4, SLC7A11, FTH1, and FTL in MC3T3-E1 cells after *S. aureus* infection. **(D)** Representative Western blot images and quantification of Runx2 in MC3T3-E1 cells infected with *S. aureus* at different MOIs. **(E)** MDA content in MC3T3-E1 cells. **(F)** Cell viability of MC3T3-E1 cells at different MOIs (n = 6). **(G)** Expressions of SLC7A11 and GPX4 in MC3T3-E1 cells infected by *S. aureus* (MOI = 50) for 4h with or without Fer-1. **(H)** Representative flow cytometry plots and quantitative analysis of the apoptosis rate in *S. aureus*-infected cells, with or without Fer-1 treatment. The apoptosis rate was evaluated using Annexin V-FITC/PI double staining. Data are presented as mean ± SD, n = 3. ns, p > 0.05, *p < 0.05, **p < 0.01, ***p < 0.001, and ****p < 0.0001.

To identify the relationship between iron metabolism and bone loss, we designed an MC3T3-E1 cell-bacteria co-culture model to mimic the PS microenvironment. We considered the optimal MOI to be 50:1 (bacteria to MC3T3-E1 cells) based on adequate survival and moderate dysfunction as reflected by CCK-8 results (**Figure 2F**). To quantify ferroptosis, characterized by lipid peroxides and iron accumulation, we used specific fluorescent probes: C11-BODIPY for lipid peroxides and FerroOrange for cytoplasmic Fe^2+^ (**Figures 2A, B**). *S. aureus* infection increased lipid peroxidation levels, Fe^2+^ content and MDA production in MC3T3-E1 cells (**Figure 2E**). Western blot analysis revealed dysregulation of ferroptosis-related proteins, as indicated by the increased expression of TfR1 and NCOA4 and decreased expression of SLC7A11, GPX4, FTH1, and FTL (**Figure 2C**). Additionally, *S. aureus* inhibited osteogenesis, as evidenced by decreased Runx-2 expression, ALP activity, and Alizarin Red staining (**Figure 2D**; **Supplementary Figures 1A, B**). Furthermore, we introduced Ferrostatin-1 (Fer-1), a selective ferroptosis inhibitor, to verify whether the observed damage and osteogenic dysfunction were specific to ferroptosis. Western blot analysis indicated that Fer-1 rescued the *S. aureus*-induced downregulation of SLC7A11 and GPX4, while flow cytometry further demonstrated that Fer-1 significantly reversed the infection-induced increase in cellular apoptosis (**Figures 2G, H**). These results indicate that ferroptosis occurs in MC3T3-E1 cells infected with *S. aureus*, however, the underlying mechanisms underlying sill unclear.

### 3.3 TfR1 relates to the ferroptosis *induced by S. aureus*

**Figure 3:**
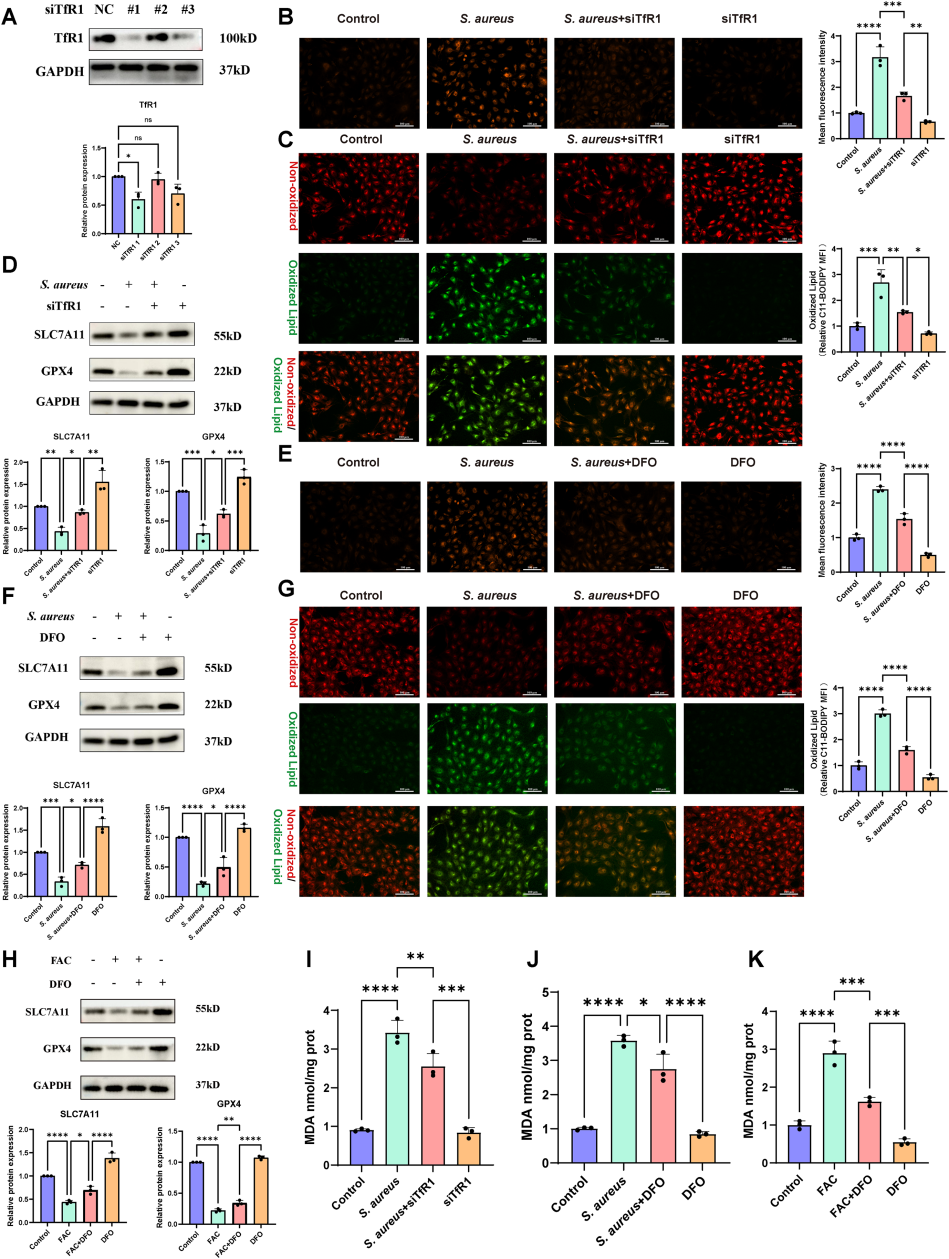
TfR1 mediates *S. aureus* induced ferroptosis and apoptosis in MC3T3-E1 cells. **(A)** Transfection efficiency of TfR1 siRNAs in MC3T3-E1 cells assessed by Western blotting. **(B)** Intracellular Fe²^+^ levels in MC3T3-E1 cells treated with *S. aureus* (MOI = 50, 4 h) with or without TfR1 siRNA. **(C)** Oxidized lipid levels in MC3T3-E1 cells mediated by TfR1 siRNA on C11-BODIPY staining fluorescent images. **(D)** Western blot analysis of SLC7A11 and GPX4 expression in MC3T3-E1 cells infected with *S. aureus* (MOI = 50, 4 h) with or without TfR1 siRNA. **(E)** Levels for ferrous ions in infected MC3T3-E1 cells with or without DFO. **(F)** Expressions of SLC7A11 and GPX4 in MC3T3-E1 cells infected by *S. aureus* with or without DFO. **(G)** Representative fluorescent images of C11-BODIPY staining for MC3T3-E1 cells mediated by DFO. **(H)** Expressions of SLC7A11 and GPX4 in MC3T3-E1 cells treated with FAC with or without DFO. **(I-K)** MDA levels in MC3T3-E1 cells under FAC treatment and *S. aureus* infection with or without DFO or TfR1 siRNA. Data are presented as mean ± SD, n = 3. ns, p > 0.05; *p < 0.05, **p < 0.01, ***p < 0.001, and ****p < 0.0001.

To precisely delineate the role of TfR1 in bacteria-induced ferroptosis in MC3T3-E1 cells, three specific siRNAs were designed to knock down TfR1. Si-TfR1–1 was the most efficient (**Figure 3A**) and significantly reversed the *S. aureus*-induced intracellular ferrous iron accumulation (**Figure 3B**). Knocking down TfR1 also reduced the accumulation of oxidized lipids (**Figure 3C**) and MDA overproduction (**Figure 3I**) caused by *S. aureus* infection. Furthermore, silencing TfR1 rescued the cells from ferroptosis by restoring the expression of anti-ferroptotic proteins SLC7A11 and GPX4 (**Figure 3D**). To confirm that iron influx drives this ferroptosis, we applied the iron chelator DFO. During *S. aureus* infection, DFO chelated the excessive intracellular ferrous iron (**Figure 3E**), effectively restored the expression of SLC7A11 and GPX4 (**Figure 3F**), and suppressed the accumulation of oxidized lipids (**Figure 3G**) and MDA (**Figure 3J**). Moreover, a standalone FAC-induced iron-overload model successfully recapitulated this ferroptotic damage. Crucially, DFO co-treatment reversed the FAC-induced downregulation of SLC7A11 and GPX4 (**Figure 3H**) and abrogated MDA overproduction (**Figure 3K**). Collectively, these results indicate that TfR1 plays an important role in mediating the iron influx and subsequent ferroptosis induced by *S. aureus* infection.

### 3.4 TfR1 inhibition rescued *S. aureus*–induced impairment of osteogenic ability in MC3T3-E1 cells

**Figure 4:**
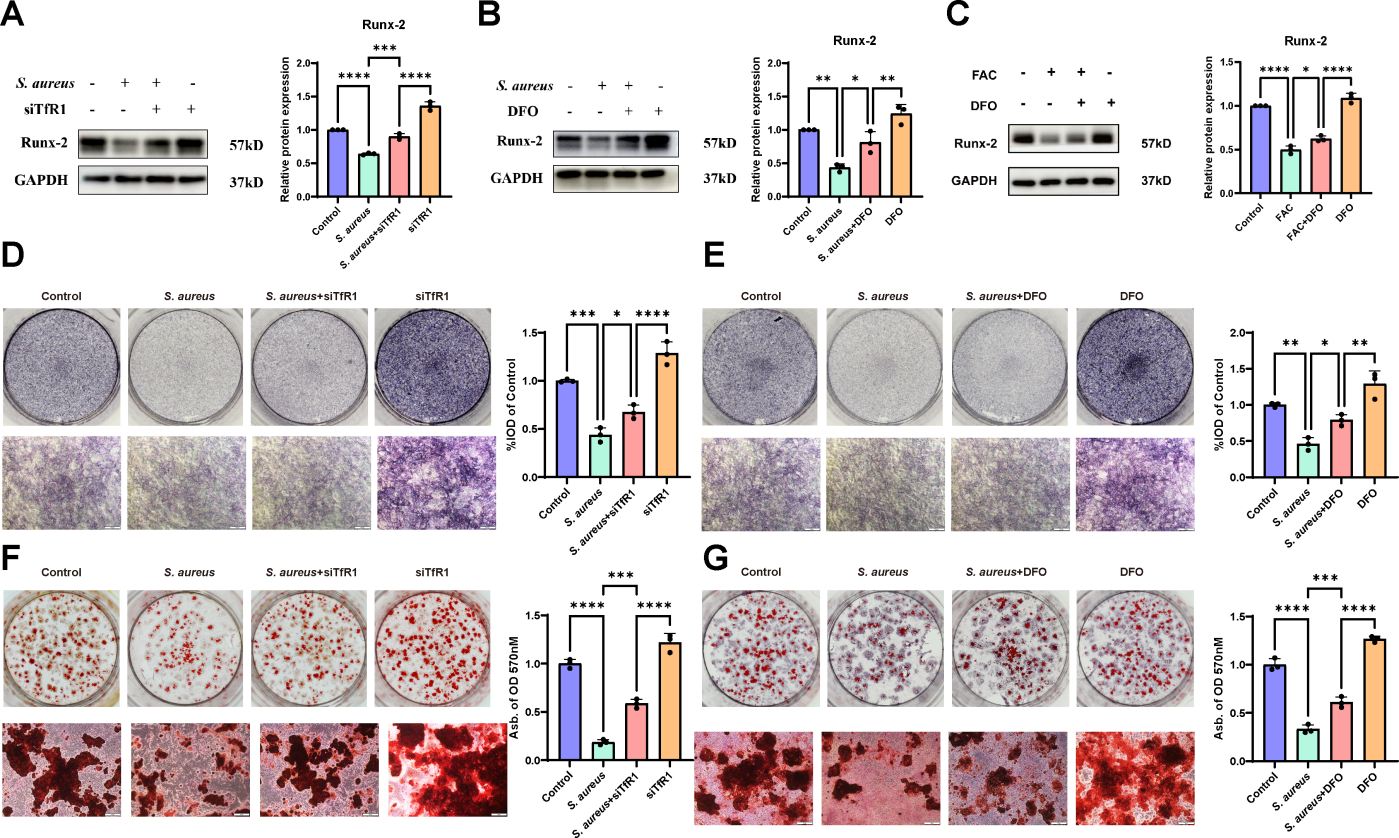
Inhibition of TfR1 rescued the osteogenic activity of MC3T3-E1 cells induced by *S. aureus*. **(A, B)** Runx2 expression in MC3T3-E1 cells infected by *S. aureus* in the presence or absence of TfR1 siRNA **(A)** or DFO **(B, C)** Expressions of Runx-2 in MC3T3-E1 cells treated by FAC with or without DFO. **(D-G)** Representative images of ALP staining (Scale bar =100μm) and Alizarin Red staining for mineralized matrix deposition in MC3T3-E1 cells (Scale bar =200μm). Both TfR1 siRNA **(D, F)** and DFO **(E, G)** can restore the osteogenic ability decreased by *S. aureus*. Data are presented as mean ± SD, n = 3. *p < 0.05, **p < 0.01, ***p < 0.001, and ****p < 0.0001.

Western blot analysis showed that silencing TfR1 and DFO treatment increased the expression of Runx-2 protein (**Figures 4A, B**). ALP and Alizarin Red staining showed the improved osteogenic ability of infected MC3T3-E1 cells (**Figures 4D–G**). Furthermore, DFO can reverse the downregulation of Runx-2 after FAC treatment via iron chelation (**Figure 4C**). These results indicate that inhibiting iron influx can ameliorate the osteogenic ability of infected MC3T3-E1 cells.

### 3.5 TfR1 mediated iron overload exacerbated mitochondrial dysfunction and apoptosis

**Figure 5:**
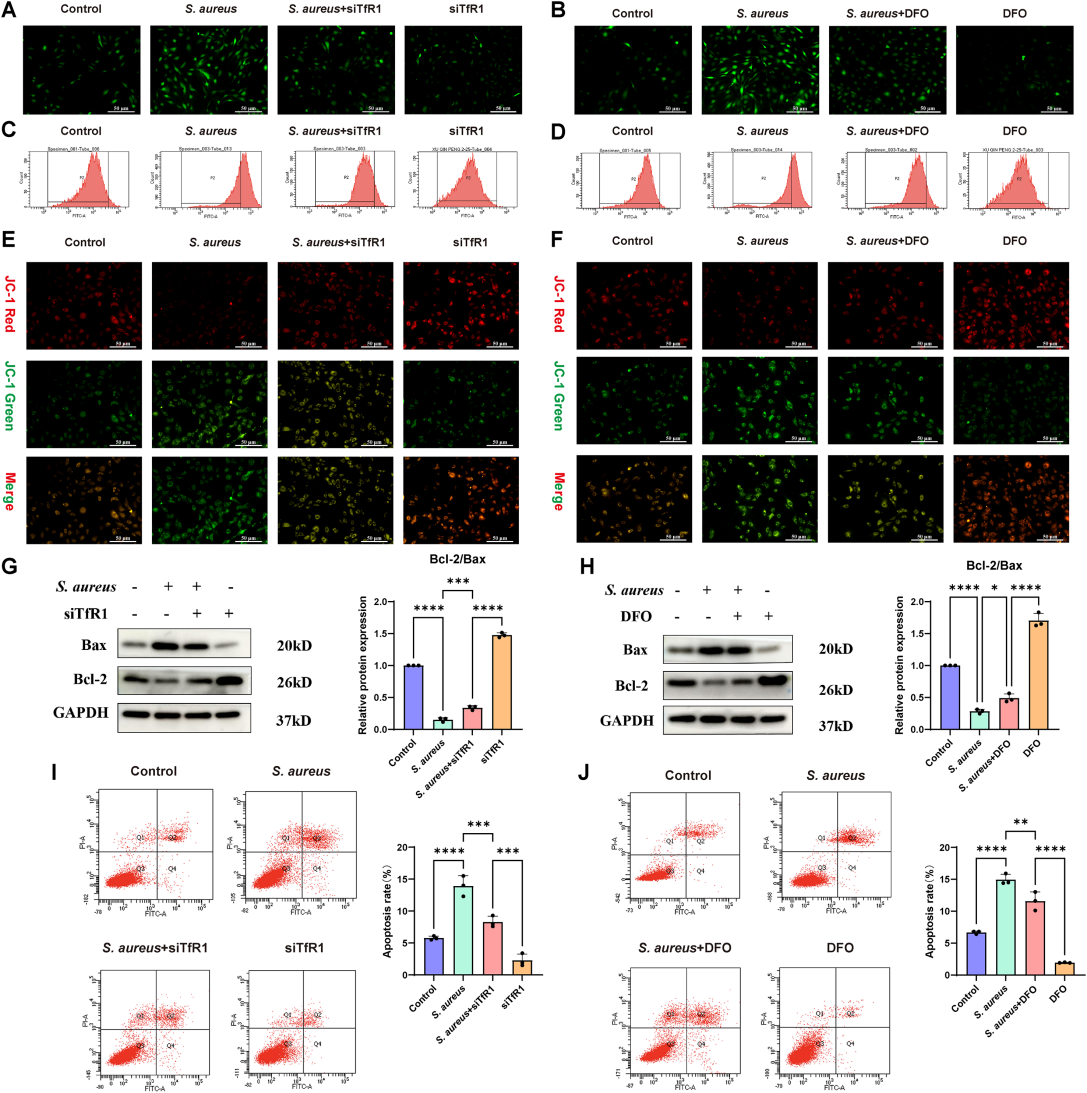
Inhibition of TfR1 alleviated *S. aureus* induced mitochondrial dysfunction and apoptosis. **(A)** TfR1 siRNA decreased intracellular ROS levels in MC3T3-E1 cells exposed to *S. aureus*, indicating by fluorescence microscopy images (Scale bar =50μm). **(B)** DFO decreased intracellular ROS levels in MC3T3-E1 cells exposed to *S. aureus*, indicating by fluorescence microscopy images (Scale bar =50μm). **(C, D)** Flow cytometric analysis labeling with DCFH-DA in in MC3T3-E1 cells intervened by siTfR1 or DFO. **(E, F)** Representative immunofluorescent images of mitochondrial membrane potential (MMP), showing red fluorescence for healthy polarized mitochondria and green fluorescence for cytosolic JC-1 monomers indicating MMP collapse (Scale bar =50μm). **(G, H)** Representative images and quantification of western blots for Bcl-2/Bax expression in MC3T3-E1 cells infected by *S. aureus* with or without TfR1 siRNA **(G)** and DFO **(H-J)** Flow cytometric analysis on MC3T3-E1 cells stained with Annexin V-FITC/PI to express the percentage of apoptotic cells. Data are presented as mean ± SD, n = 3. *p < 0.05, **p < 0.01, ***p < 0.001, and ****p < 0.0001.

Recent studies have shown that iron overload can result in a series of downstream effects after *S. aureus* infection, including oxidative stress, mitochondrial dysfunction, and cellular apoptosis (26). Thus, we examined whether TfR1 siRNA and DFO could affect ROS production and mitochondrial function. Immunofluorescence analysis showed that both TfR1 siRNA and DFO significantly decreased the intracellular levels of ROS (**Figures 5A, B**). Flow cytometric analysis revealed consistent changes (**Figures 5C, D**; **Supplementary Figures 2A, B**). Mitochondrial dysfunction was can also inferred from alterations in MMP and JC-1 monomer levels (**Figures 5E, F**). *S. aureus* contains various cell toxins related to apoptosis (27). Western blotting presented an increased ratio of Bax to Bcl-2, indicating *S. aureus*-induced apoptosis (**Supplementary Figure 2C**), while TfR1 siRNA and DFO reverse it (**Figures 5G, H**). Annexin V/PI staining in flow cytometry demonstrated that TfR1 siRNA and DFO protected MC3T3-E1 cells from apoptosis triggered by *S. aureus* (**Figures 5I, J**).

### 3.6 *S. aureus* infection induces pyroptosis via the cGAS/STING pathway

**Figure 6:**
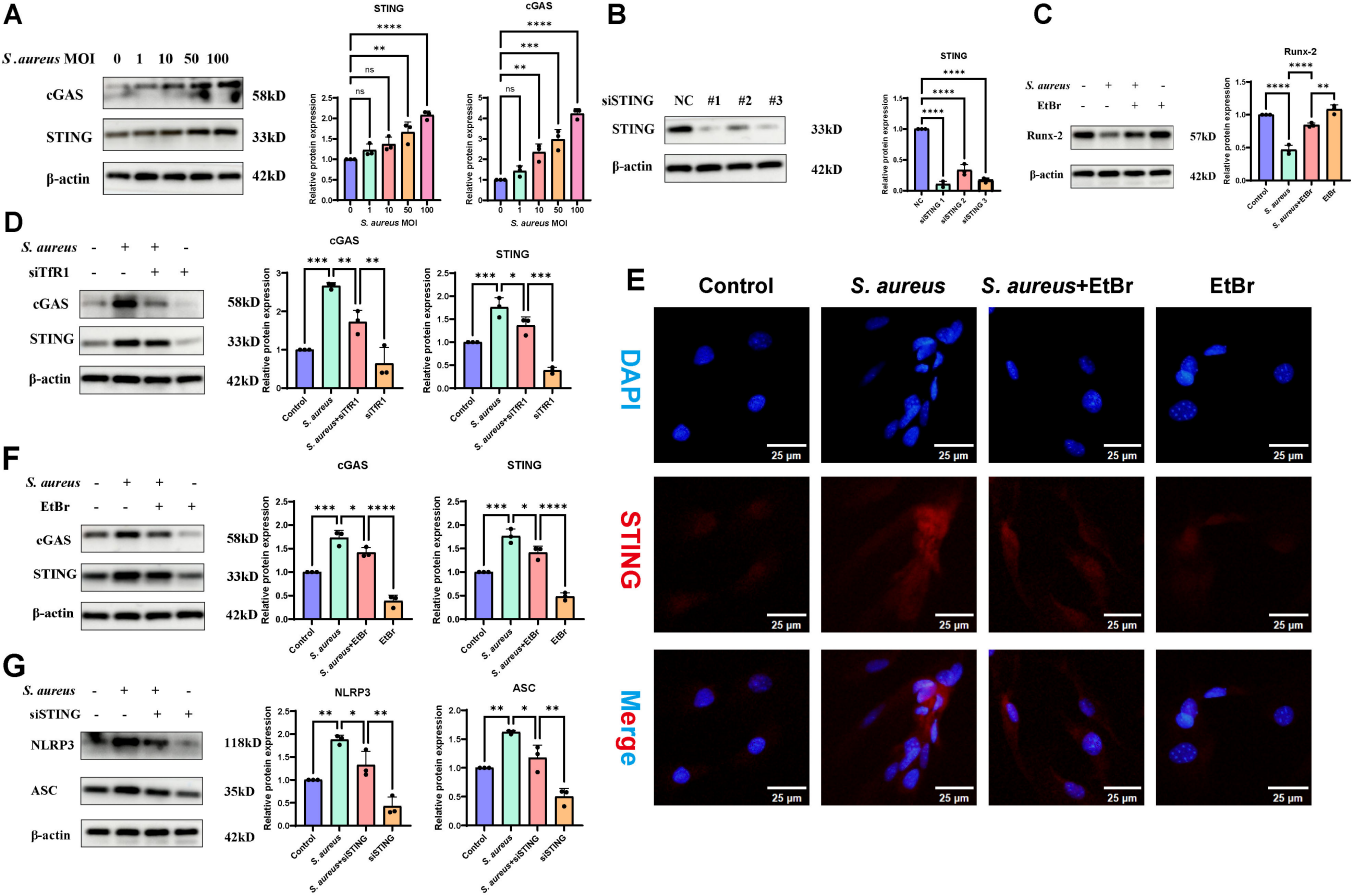
Activation of the mtDNA/cGAS/STING pathway in MC3T3-E1 cells infected by *S. aureus.* **(A)** Representative images and quantification of cGAS and STING in MC3T3-E1 cells infected by *S. aureus* at different MOIs. **(B)** Transfection efficiency of different STING siRNAs (NC, #1, #2, #3) in MC3T3-E1 cells. **(C)** Expressions of Runx-2 in MC3T3-E1 cells infected by *S. aureus* with or without EtBr. **(D)** Representative images and quantification of cGAS and STING expression in MC3T3-E1 cells infected by *S. aureus* with or without TfR1 siRNA. **(E)** Representative immunofluorescent images of STING in MC3T3-E1 cells treated with or without EtBr. **(F)** Expressions of cGAS and STING in MC3T3-E1 cells infected by *S. aureus* with or without EtBr. **(G)** Western blots and quantification for NLRP3 and ASC in MC3T3-E1 cells infected by *S. aureus* with or without STING siRNA. Data are presented as mean ± SD, n = 3. *p < 0.05, **p < 0.01, ***p < 0.001, and ****p < 0.0001.

Recent studies show that cells may experience pyroptosis and apoptosis via the cGAS-STING pathway in various diseases (28). As shown in **Figure 6A**, the expression levels of cGAS and STING increased in an MOI-dependent manner. Our results showed that TfR1 siRNA inhibited the cGAS-STING activity induced by *S. aureus* (**Figure 6D**). Mitochondria are the primary site of cellular iron metabolism, and mitochondrial dysfunction plays a crucial role in the progression of inflammation (29). Previous studies have shown that mitochondrial damage and the release of mitochondrial DNA (mtDNA) into the cytoplasm are crucial to inflammatory reactions and cellular damage (30). To evaluate the role of mtDNA in STING activation in response to *S. aureus*, we introduced ethidium bromide (EtBr) to deplete mtDNA in MC3T3-E1 cells. Western blotting and immunofluorescence staining showed that EtBr inhibited the *S. aureus*-induced STING activation (**Figures 6E, F**) and restored the impaired osteogenic ability of MC3T3-E1 cells (**Figure 6C**). We selected STING siRNA to investigate the role of the cGAS-STING pathway in PS (**Figure 6B**). STING knockdown reduced the expression of pyroptotic NLRP3 and ASC (**Figure 6G**). These findings suggest that TfR1 mediates PS inflammation and further causing pyroptosis via mtDNA release and cGAS-STING pathway activation.

### 3.7 Inhibition of TfR1 reduces the expression of inflammatory factors in infection

**Figure 7:**
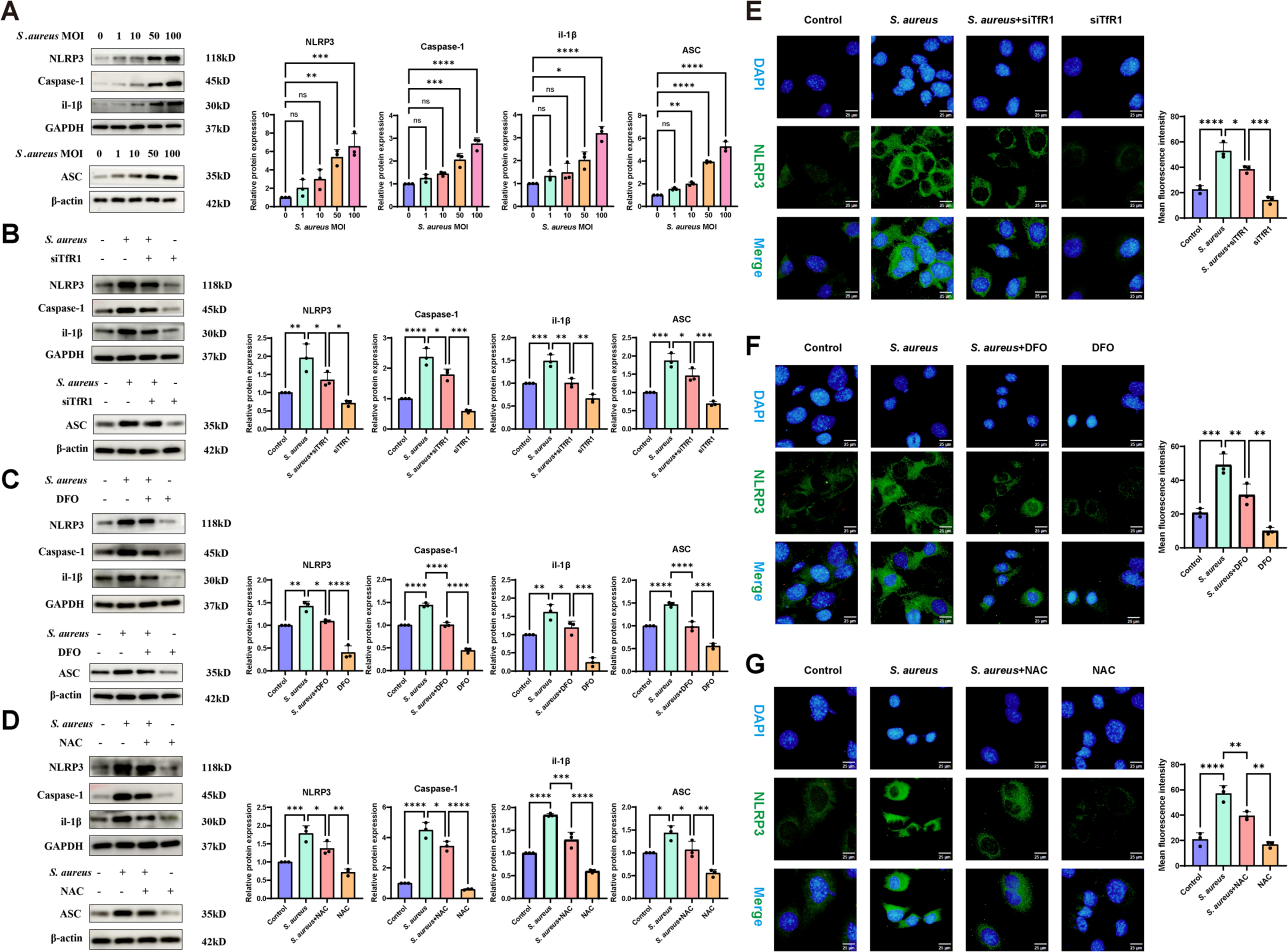
Inhibition of TfR1 alleviates pyroptosis induced by *S. aureus*. **(A)** Expressions of NLRP3, Caspase-1, IL-1β and ASC in MC3T3-E1 cells under different MOIs. **(B, C)** TfR1 siRNA and DFO decreased expressions of pyroptotic proteins in MC3T3-E1 cells. **(D)** Expressions of NLRP3, Caspase-1, IL-1β, and ASC in MC3T3-E1 cells infected by *S. aureus* with or without NAC. **(E-G)** Representative immunofluorescent images of NLRP3 in MC3T3-E1 cells treated with or without TfR1 siRNA **(E)**, DFO **(F)** and NAC **(G)** (Scale bar = 25μm). Data are presented as mean ± SD, n = 3. ns, p > 0.05; *p < 0.05, **p < 0.01, ***p < 0.001, and ****p < 0.0001.

Previous studies showed that ferroptosis could promote the expression of intracellular inflammatory factors (31, 32), but the mechanism remained unclear. *S. aureus* enhanced the expression of inflammatory proteins such as NLRP3, Caspase-1, IL-1β and ASC in MC3T3-E1 cells (**Figure 7A**), which was also reversed upon exposure to TfR1 siRNA or DFO (**Figures 7B, C**). After treatment with N-acetylcysteine (NAC), a well-established antioxidant thiol compound, increases intracellular glutathione content (33), the MC3T3-E1 cells showed reduced the expression of pyroptotic proteins and inflammatory cytokines (**Figure 7D**). Immunofluorescence staining showed that the upregulation of NLRP3 was attenuated by TfR1 siRNA, DFO, or NAC in the *S. aureus-*infecting environment (**Figures 7E–G**).

### 3.8 Inhibition of TfR1 could alleviate the osteoclastsogenesis induced by *S. aureus*

**Figure 8:**
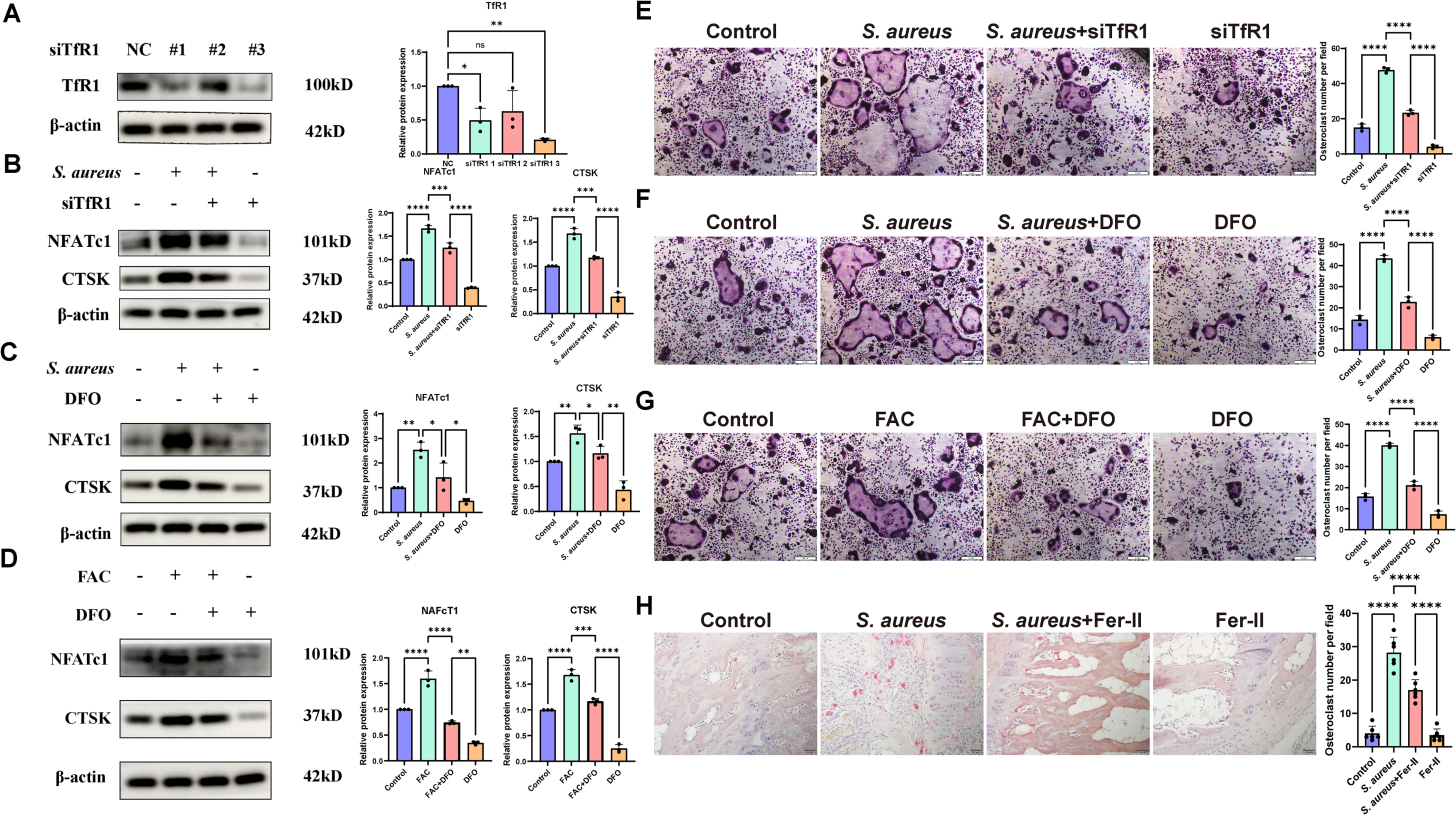
Inhibition of TfR1 can reduce the activation of osteoclasts induced by *S. aureus*. **(A)** Transfection efficiency of different TfR1 siRNA in osteoclasts assessed by western blot analysis. **(B, C)** Expressions of NFATc1 and CTSK in osteoclast infected by *S. aureus* regulated by TfR1 siRNA **(B)** or DFO **(C, D)** Expressions of NFATc1 and CTSK in osteoclasts treated with FAC with or without DFO. **(E-G)** TRAP staining showing that the osteoclastic differentiation induced by *S. aureus* or FAC can be alleviated by TfR1 knockdown **(E)** and iron chelator DFO **(F, G)** (Scale bar = 200μm). **(H)** Representative TRAP staining images and quantification of osteoclast number per field in vertebral tissues from the Control, *S. aureus*, *S. aureus* + Fer-II, and Fer-II groups. Data are presented as mean ± SD, n = 3. ns, p > 0.05; *p < 0.05, **p < 0.01, ***p < 0.001, and ****p < 0.0001.

Bone homeostasis depends on bone formation by osteoblasts and bone resorption by osteoclasts (34). However, unlike its effects on osteoblasts, a previous study demonstrated that iron overload promotes osteoclast formation and function (35). Thus, we investigated whether TfR1-mediated iron overload participates in *S. aureus*-induced osteoclastogenesis. All three siRNAs targeting TfR1 (si-TfR1-1, si-TfR1-2, and si-TfR1-3) reduced the expression of TfR1, with si-TfR1–3 showing the highest knockdown efficiency (**Figure 8A**). Both TfR1-3 and DFO alleviated the expression of NFATc1 and CTSK (**Figures 8B, C**). TRAP staining showed that TfR1 siRNA and DFO attenuated osteoclastic overactivation triggered *S. aureus* (**Figures 8E, F**). Moreover, *in vivo* osteoclastogenesis is activated by *S. aureus* in infected vertebrae, which was significantly suppressed by the TfR1 inhibitor Fer-II (**Figure 8H**). To further confirm whether iron overload primarily drives osteoclastogenesis in infection, we established an independent iron overload model by treating BMMs with FAC. Consistent with the trends in the *S. aureus*-infected vertebrae, FAC treatment upregulated the expression of NFATc1 and CTSK (**Figure 8D**) and promoted osteoclastogenesis as evidenced by TRAP staining (**Figure 8G**). Similarly, DFO effectively reversed the osteoclastogenesis induced by the FAC treatment.

### 3.9 Inhibiting TfR1 can ameliorate the bone loss in pyogenic spondylitis

**Figure 9:**
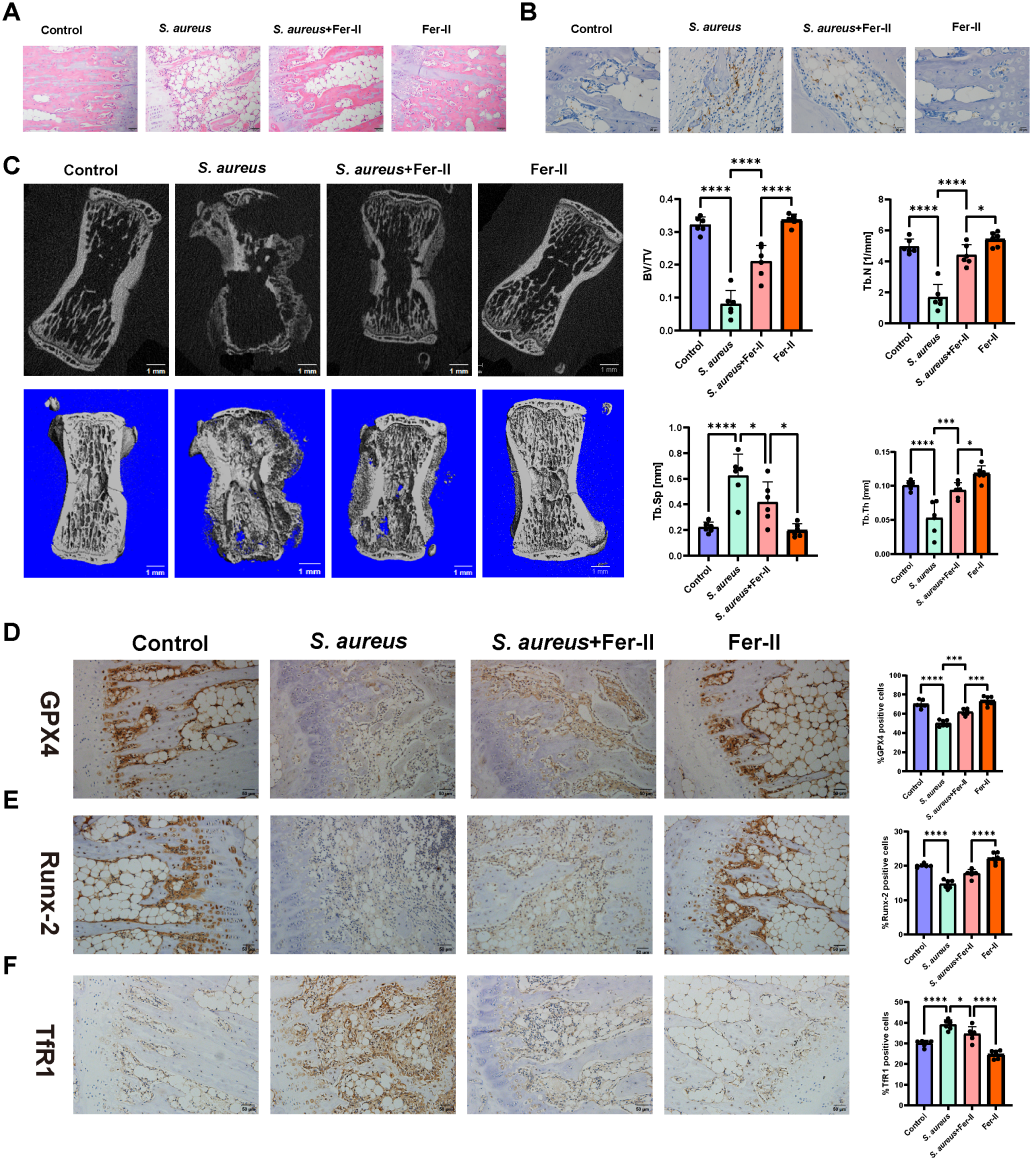
Inhibiting TfR1 ameliorated bone loss in pyogenic spondylitis. **(A)** HE staining showed bone destruction in the control, *S. aureus*, *S. aureus* + Fer-II and Fer-II groups (Scale bar = 50μm). **(B)** Dab-enhanced Prussian blue staining, indicating iron content in the control, *S. aureus*, *S. aureus* + Fer-II and Fer-II groups (Scale bar = 20μm). **(C)** Micro-CT analysis of trabecular bone destruction in the control, *S. aureus*, *S. aureus* + Fer-II and Fer-II groups (Scale bar = 1mm). **(D)** Immunohistochemistry for ferroptotic marker GPX4 in four groups (Scale bar = 50μm). **(E)** Representative immunohistochemistry images and quantification of Runx-2 in four groups(Scale bar = 50μm). **(F)** Immunohistochemical staining of TfR1 from the control, *S. aureus*, *S. aureus* + Fer-II and Fer-II groups(Scale bar = 50μm). Data are presented as mean ± SD, n = 6. *p < 0.05, **p < 0.01, ***p < 0.001, and ****p < 0.0001.

Fer-II, a novel and selective TfR1 inhibitor, has been shown to substantially suppress TfR1 activity both *in vivo* and *in vitro* (36). We injected planktonic *S. aureus* into the vertebrae of rats to mimic pyogenic spondylitis (20) and to evaluate the protective effect of Fer-II. Following surgery, rats were intraperitoneally administered 10 mg/kg Fer-II once every two days (21). HE staining and micro-CT scanning showed that Fer-II alleviated bone loss caused by *S. aureus* infection (**Figures 9A, C**). Dab-enhanced Prussian blue staining showed that Fer-II reduced iron overload caused by *S. aureus* (**Figure 9B**). Immunohistochemical staining revealed that Fer-II administration significantly inhibited TfR1 expression (**Figure 9F**) and increased the expression of GPX4 and Runx-2 (**Figures 9D, E**). These data demonstrate that the Fer-II can attenuate infection-induced bone loss.

## 4 Discussion

Iron is the most abundant trace element in humans and is crucial for cellular metabolism and function. Accumulating evidence has demonstrated abnormal iron deposition in various orthopedic diseases, including osteoporosis and osteomyelitis (8, 37). Iron overload has been increasingly recognized as a critical contributor to infection-associated bone loss (7, 38). PS is a severe infectious bone disease predominantly caused by *S. aureus*, characterized by rapid bone destruction driven by bacterial colonization and persistent inflammatory responses. However, the precise molecular mechanisms underlying infection-induced bone loss remain incompletely understood. Therefore, elucidating the role of dysregulated iron metabolism in the pathogenesis of PS is significant. In the present study, we demonstrated that iron overload occurs in both osteoblasts and osteoclasts, triggering a series of pathological signaling cascades, ultimately leading to deteriorated bone loss in PS. These findings highlight a potential therapeutic target of PS.

TfR1 functions as a primary regulator of cellular iron uptake and plays a central role in maintaining iron homeostasis, while also contributing to the pathogenesis of multiple diseases (39). In this study, we observed abnormal iron accumulation accompanied by aberrant upregulation of TfR1 expression both *in vivo* and *in vitro*. Excess intracellular iron promoted lipid peroxidation, thereby inducing cellular damage and ferroptosis. Previous studies have reported the involvement of ferroptosis in bacterial infections (10). Consistently, we detected elevated lipid peroxidation levels in *S. aureus*–infected MC3T3-E1 cells. Moreover, ferroptosis, characterized by the downregulation of GPX4, SLC7A11, FTH1, and FTL, resulted in impaired osteogenic capacity and contributed to osteoporosis-like phenotypes. Although *S. aureus* infection is known to impair in MC3T3-E1 cells (40), the underlying mechanism remains unexplained. Our findings demonstrate that TfR1-mediated iron overload induces ferroptosis, providing new mechanistic insights into infection-associated osteogenic impairment.

Mitochondria are the central organelles involving cellular iron metabolism (41). Iron serves as a key catalyst for ROS generation, and disruption of iron homeostasis leads to excessive ROS production, oxidative stress, and tissue injury (42). Our results demonstrated that iron overload in MC3T3-E1 cells induced mitochondrial structural damage, enhanced ROS production, and subsequent cellular injury. Knocking TfR1 down restored mitochondrial integrity, reduced ROS levels, and attenuated ferroptosis induced by iron overload. Inhibition of TfR1-mediated iron influx significantly reduced intracellular iron accumulation, alleviated oxidative stress, and improved mitochondrial function. DFO, a clinically approved iron chelator used for iron overload disorders such as thalassemia and sickle cell disease (43), exerted protective effects in MC3T3-E1 cells, suggesting its potential translational application in preventing infection-related bone loss.

Our data further indicate that iron-induced mitochondrial dysfunction activates the cGAS-STING signaling pathway in *S. aureus*–infected MC3T3-E1 cells. Pharmacological inhibition of mtDNA release using EtBr markedly suppressed cGAS-STING activation and restored osteogenic function. These findings suggest that mitochondrial dysfunction–driven mtDNA release plays a pivotal role in coupling iron dysregulation to innate immune activation. Emerging evidence indicates that the cGAS-STING pathway can activate NF-κB during bacterial infection, which in turn promotes NLRP3 inflammasome activation (44), leading to excessive production of pro-inflammatory cytokines and inflammatory cell death. These results establish a mechanistic link between ferroptosis, pyroptosis, and apoptosis in infected osteoblasts.

Pyroptosis is an inflammatory form of lytic programmed cell death mediated by the gasdermin family of proteins (45). Previous studies showed that ferroptosis promotes the expression of inflammatory mediators and indicated the potential mechanistic link between ferroptosis and pyroptosis (46). Ferroptotic cells release damage-associated molecular patterns and alarmins, which are sensed by pattern recognition receptors (PRRs), thereby triggering inflammatory cell recruitment and cytokine release (47). In our models, *S. aureus* infection induced typical pyroptosis, as evidenced by increased expression of NLRP3, Caspase-1, IL-1β, and ASC. Apoptotic signaling appeared to occur subsequent to pyroptosis (48). Our findings indicate a sequential rather than parallel relationship among ferroptosis, pyroptosis, and apoptosis, where ferroptosis acts as the primary initiating trigger. Theoretically, TfR1-mediated iron overload induces severe ROS accumulation and mitochondrial damage, which subsequently activates the downstream intrinsic apoptotic cascade. This is also evidenced by our findings that inhibiting iron influx (via TfR1 siRNA or DFO) attenuates not only ferroptosis but also pyroptotic and apoptotic markers such as the Bax/Bcl-2 ratio. Therefore, the observed apoptosis is a secondary consequence driven by ferroptosis-induced oxidative stress and mitochondrial dysfunction. However, the possibility that bacterial cytotoxins induce apoptosis cannot be totally excluded (27).

Osteoclasts are specialized bone-resorbing cells essential for physiological bone remodeling and the pathogenesis of skeletal diseases (49). Previous studies have demonstrated that iron overload promotes osteoclast differentiation and functional activation (50). Consistent with these findings, we observed that *S. aureus* infection enhanced osteoclast formation and activity, which was largely attributable to TfR1-mediated iron influx. Similarly, FAC significantly promoted osteoclast differentiation and function. Both TfR1 silencing and DFO treatment attenuated osteoclast activation, suggesting a critical role for iron homeostasis in osteoclast overactivation during PS (51). Given the high iron demand during osteoclastogenesis, infection-induced iron accumulation may exacerbate bone loss. Fer-II, a novel and selective TfR1 inhibitor, promotes TfR1 degradation and effectively suppresses ferroptosis *in vivo* and *in vitro* (36). Our animal experiments also demonstrated that Fer-II administration alleviated ferroptosis and protected against *S. aureus*-induced bone loss, highlighting its potential therapeutic value in preserving bone homeostasis in infectious bone diseases. However, it is important to acknowledge that physiological bone remodeling fundamentally relies on the coupling between bone-forming and bone-resorbing cells (52). While our current study primarily focused on the direct intracellular effects of iron overload on individual bone cell types, the complex inflammatory PS microenvironment likely also disrupts the dynamic intercellular crosstalk. Evaluating the impact of iron dysregulation on this osteoblast-osteoclast communication presents a critical avenue for future research.

## 5 Conclusions

Bone loss in PS is driven by the coordinated processes of ferroptosis, pyroptosis, and apoptosis in osteoblasts, alongside the aberrant activation of osteoclasts. These pathological processes can be effectively attenuated by inhibiting TfR1-mediated iron influx. Our findings identify TfR1 as a promising therapeutic target for the prevention and treatment infection-associated bone loss.

## 6 Limitations

This study has several limitations. First, this study elucidates the mechanisms of bone loss in *S. aureus*-induced PS, these results may not be generalizable to other forms of PS and require broader validation. Second, bone loss is mediated by complex and multifactorial pathways, and iron overload represents only one of the potential mechanisms contributing to pyroptosis and apoptosis. Finally, the molecular mechanisms governing osteoclast activation in this context remain incompletely defined and warrant further investigation.

## Data Availability

The original contributions presented in the study are included in the article/**Supplementary Material**. Further inquiries can be directed to the corresponding authors.

## Supplementary material

Supplementary Figure 1 *S. aureus* inhibited MC3T3-E1 cells osteogenic differentiation through inducing ferroptosis. **(A)** ALP staining for MC3T3-E1 cells under different MOI. (Scale bar =200μm). Semi-quantitative analysis indicated the damaged osteogenesis. **(B)** Alizarin Red staining for calcium deposition and its semi-quantitative analysis under diverse MOI in MC3T3-E1 cells (Scale bar =100μm). Data are presented as mean ± SD, n = 3. ***p < 0.001, and ****p < 0.0001.

Supplementary Figure 2 Inhibition of TfR1 alleviated *S. aureus* induced mitochondrial dysfunction and apoptosis. **(A, B)** Flow cytometric analysis was conducted to quantify the ROS production. **(C)** Western blot for Bax and Bcl-2 protein under different MOI. Data are presented as mean ± SD, n = 3. ns, p > 0.05, *p < 0.05, **p < 0.01, ***p < 0.001, and ****p < 0.0001.

## References

[1] Sato K Yamada K Yokosuka K Yoshida T Goto M Matsubara T . Pyogenic spondylitis: Clinical features, diagnosis and treatment. Kurume Med J. (2019) 65:83–9. doi: 10.2739/kurumemedj.MS653001 31406038

[2] Kim NJ . Microbiologic diagnosis of pyogenic spondylitis. Infect Chemother. (2021) 53:238–46. doi: 10.3947/ic.2021.0054 34216118 PMC8258299

[3] Liu X Zheng M Sun J Cui X . A diagnostic model for differentiating tuberculous spondylitis from pyogenic spondylitis on computed tomography images. Eur Radiol. (2021) 31:7626–36. doi: 10.1007/s00330-021-07812-1 33768287

[4] Rawall S Hiatt LA Rajaram SM Theiss S . Management of pyogenic spondylodiscitis in adults. J Am Acad Orthop Surg. (2025) 33:1257–64. doi: 10.5435/JAAOS-D-24-01088 40279562

[5] Hao M Jiang Y Zhang Y Yang X Han J . Ferroptosis regulation by methylation in cancer. Biochim Biophys Acta Rev Cancer. (2023) 1878:188972. doi: 10.1016/j.bbcan.2023.188972 37634887

[6] Tang D Chen X Kang R Kroemer G . Ferroptosis: molecular mechanisms and health implications. Cell Res. (2021) 31:107–25. doi: 10.1038/s41422-020-00441-1 33268902 PMC8026611

[7] Zhou S-R Li W-G Yang L-D Xiang H Jin Y Feng J-B . PTGS2 silencing inhibits ferroptosis in Staphylococcus aureus-induced osteomyelitis by blocking the IL-17A signaling pathway. Inflammation. (2025) 48:3841–57. doi: 10.1007/s10753-025-02296-3 40257651 PMC12722496

[8] Shi X Tang L Ni H Li M Wu Y Xu Y . Identification of ferroptosis-related biomarkers for diagnosis and molecular classification of Staphylococcus aureus-induced osteomyelitis. J Inflammation Res. (2023) 16:1805–23. doi: 10.2147/JIR.S406562 37131411 PMC10149083

[9] Christmas BAF Rolfe MD Rose M Green J . Staphylococcus aureus adaptation to aerobic low-redox-potential environments: implications for an intracellular lifestyle. Microbiol (Read). (2019) 165:779–91. doi: 10.1099/mic.0.000809 31100054

[10] Soe YM Bedoui S Stinear TP Hachani A . Intracellular Staphylococcus aureus and host cell death pathways. Cell Microbiol. (2021) 23:e13317. doi: 10.1111/cmi.13317 33550697

[11] Gao S Gao Y Cai L Qin R . Luteolin attenuates Staphylococcus aureus-induced endometritis through inhibiting ferroptosis and inflammation via activating the Nrf2/GPX4 signaling pathway. Microbiol Spectr. (2024) 12:e0327923. doi: 10.1128/spectrum.03279-23 38169293 PMC10846197

[12] Zhao L Jin L Yang B . Diosmetin alleviates S. aureus-induced mastitis by inhibiting SIRT1/GPX4 mediated ferroptosis. Life Sci. (2023) 331:122060. doi: 10.1016/j.lfs.2023.122060 37652155

[13] Liu X Zheng M Jiang Z Wang G Li T Sun J . Computed tomography imaging characteristics help to differentiate pyogenic spondylitis from brucellar spondylitis. Eur Spine J. (2020) 29:1490–8. doi: 10.1007/s00586-019-06214-8 31754822

[14] Li T Liu T Jiang Z Cui X Sun J . Diagnosing pyogenic, brucella and tuberculous spondylitis using histopathology and MRI: A retrospective study. Exp Ther Med. (2016) 12:2069–77. doi: 10.3892/etm.2016.3602 27698694 PMC5038492

[15] Wang W Ma Z Feng X Ren J Sun S Shao Y . TfR1 mediated iron metabolism dysfunction as a potential therapeutic target for osteoarthritis. Arthritis Res Ther. (2024) 26:71. doi: 10.1186/s13075-024-03304-x 38493104 PMC10943767

[16] Gammella E Buratti P Cairo G Recalcati S . The transferrin receptor: the cellular iron gate. Metallomics. (2017) 9:1367–75. doi: 10.1039/c7mt00143f 28671201

[17] Mietto BS Jhelum P Schulz K David S . Schwann cells provide iron to axonal mitochondria and its role in nerve regeneration. J Neurosci. (2021) 41:7300–13. doi: 10.1523/JNEUROSCI.0900-21.2021 34272312 PMC8387113

[18] Kawabata H . Transferrin and transferrin receptors update. Free Radic Biol Med. (2019) 133:46–54. doi: 10.1016/j.freeradbiomed.2018.06.037 29969719

[19] Jiang Z Wang H Qi G Jiang C Chen K Yan Z . Iron overload‐induced ferroptosis of osteoblasts inhibits osteogenesis and promotes osteoporosis: an *in vitro* and *in vivo* study. IUBMB Life. (2022) 74:1052–69. doi: 10.1002/iub.2656 35638167

[20] Gamada H Funayama T Setojima Y Ogata Y Sunami T Sakashita K . Posterior fixation without debridement for pyogenic spondylodiscitis can promote infection control: initial evaluation of a pyogenic spondylodiscitis posterior fixation rat model. Eur Spine J. (2025) 34:2165–75. doi: 10.1007/s00586-025-08750-y 40029355

[21] Cheng Y Qu W Li J Jia B Song Y Wang L . Ferristatin II, an iron uptake inhibitor, exerts neuroprotection against traumatic brain injury via suppressing ferroptosis. ACS Chem Neurosci. (2022) 13:664–75. doi: 10.1021/acschemneuro.1c00819 35143157

[22] Shi Z Yang F Pang Q Hu Y Wu H Yu X . The osteogenesis and the biologic mechanism of thermo-responsive injectable hydrogel containing carboxymethyl chitosan/sodium alginate nanoparticles towards promoting osteal wound healing. Int J Biol Macromol. (2023) 224:533–43. doi: 10.1016/j.ijbiomac.2022.10.142 36265540

[23] Hoeft K Bloch DB Graw JA Malhotra R Ichinose F Bagchi A . Iron loading exaggerates the inflammatory response to the toll-like receptor 4 ligand lipopolysaccharide by altering mitochondrial homeostasis. Anesthesiology. (2017) 127:121. doi: 10.1097/ALN.0000000000001653 28430694 PMC5478432

[24] Krauss JL Roper PM Ballard A Shih C-C Fitzpatrick JAJ Cassat JE . Staphylococcus aureus infects osteoclasts and replicatesintracellularly. mBio. (2019) 10:e02447–9. doi: 10.1128/mBio.02447-19 31615966 PMC6794488

[25] Dou C Li J Kang F Cao Z Yang X Jiang H . Dual effect of cyanidin on RANKL-induced differentiation and fusion of osteoclasts. J Cell Physiol. (2016) 231:558–67. doi: 10.1002/jcp.24916 25545964

[26] Liu K Zhou X Fang L Dong J Cui L Li J . PINK1/parkin-mediated mitophagy alleviates Staphylococcus aureus-induced NLRP3 inflammasome and NF-κB pathway activation in bovine mammary epithelial cells. Int Immunopharmacol. (2022) 112:109200. doi: 10.1016/j.intimp.2022.109200 36063687

[27] Zhang X Hu X Rao X . Apoptosis induced by Staphylococcus aureus toxins. Microbiol Res. (2017) 205:19–24. doi: 10.1016/j.micres.2017.08.006 28942840

[28] Xu Y Chen C Liao Z Xu P . cGAS-STING signaling in cell death: Mechanisms of action and implications in pathologies. Eur J Immunol. (2023) 53:e2350386. doi: 10.1002/eji.202350386 37424054

[29] Newman LE Shadel GS . Mitochondrial DNA release in innate immune signaling. Annu Rev Biochem. (2023) 92:299–332. doi: 10.1146/annurev-biochem-032620-104401 37001140 PMC11058562

[30] Jiménez-Loygorri JI Villarejo-Zori B Viedma-Poyatos Á Zapata-Muñoz J Benítez-Fernández R Frutos-Lisón MD . Mitophagy curtails cytosolic mtDNA-dependent activation of cGAS/STING inflammation during aging. Nat Commun. (2024) 15:830. doi: 10.1038/s41467-024-45044-1 38280852 PMC10821893

[31] Wang W Jing X Du T Ren J Liu X Chen F . Iron overload promotes intervertebral disc degeneration via inducing oxidative stress and ferroptosis in endplate chondrocytes. Free Radic Biol Med. (2022) 190:234–46. doi: 10.1016/j.freeradbiomed.2022.08.018 35981695

[32] Sun Y Chen P Zhai B Zhang M Xiang Y Fang J . The emerging role of ferroptosis in inflammation. BioMed Pharmacother. (2020) 127:110108. doi: 10.1016/j.biopha.2020.110108 32234642

[33] Guo L Zhang H Li W Zhan D Wang M . N-acetyl cysteine inhibits lipopolysaccharide-mediated induction of interleukin-6 synthesis in MC3T3-E1 cells through the NF-kB signaling pathway. Arch Oral Biol. (2018) 93:149–54. doi: 10.1016/j.archoralbio.2018.06.007 29929056

[34] Chen X Wang Z Duan N Zhu G Schwarz EM Xie C . Osteoblast-osteoclast interactions. Conn Tissue Res. (2018) 59:99–107. doi: 10.1080/03008207.2017.1290085 28324674 PMC5612831

[35] Che J Yang J Zhao B Zhang G Wang L Peng S . The effect of abnormal iron metabolism on osteoporosis. Biol Trace Elem Res. (2020) 195:353–65. doi: 10.1007/s12011-019-01867-4 31473898

[36] Byrne SL Buckett PD Kim J Luo F Sanford J Chen J . Ferristatin II promotes degradation of transferrin receptor-1 *in vitro* and *in vivo*. PloS One. (2013) 8:e70199. doi: 10.1371/journal.pone.0070199 23894616 PMC3720890

[37] Gao Z Chen Z Xiong Z Liu X . Ferroptosis - A new target of osteoporosis. Exp Gerontol. (2022) 165:111836. doi: 10.1016/j.exger.2022.111836 35598699

[38] Qin H-J He S-Y Shen K Lin Q-R Hu Y-J Chen Z-L . Melatonin, a potentially effective drug for the treatment of infected bone nonunion. J Pineal Res. (2024) 76:e12914. doi: 10.1111/jpi.12914 37753741

[39] Fillebeen C Charlebois E Wagner J Katsarou A Mui J Vali H . Transferrin receptor 1 controls systemic iron homeostasis by fine-tuning hepcidin expression to hepatocellular iron load. Blood. (2019) 133:344–55. doi: 10.1182/blood-2018-05-850404 30538134

[40] Mouton W Josse J Jacqueline C Abad L Trouillet-Assant S Caillon J . Staphylococcus aureus internalization impairs osteoblastic activity and early differentiation process. Sci Rep. (2021) 11:17685. doi: 10.1038/s41598-021-97246-y 34480054 PMC8417294

[41] Paul BT Manz DH Torti FM Torti SV . Mitochondria and iron: current questions. Expert Rev Hematol. (2017) 10:65–79. doi: 10.1080/17474086.2016.1268047 27911100 PMC5538026

[42] Dickson KB Zhou J . Role of reactive oxygen species and iron in host defense against infection. Front Biosci (Landm Ed). (2020) 25:1600–16. doi: 10.2741/4869 32114446

[43] Jeney V . Clinical impact and cellular mechanisms of iron overload-associated bone loss. Front Pharmacol. (2017) 8:77. doi: 10.3389/fphar.2017.00077 28270766 PMC5318432

[44] Zhang K Huang Q Li X Zhao Z Hong C Sun Z . The cGAS-STING pathway in viral infections: a promising link between inflammation, oxidative stress and autophagy. Front Immunol. (2024) 15:1352479. doi: 10.3389/fimmu.2024.1352479 38426093 PMC10902852

[45] Yang F Bettadapura SN Smeltzer MS Zhu H Wang S . Pyroptosis and pyroptosis-inducing cancer drugs. Acta Pharmacol Sin. (2022) 43:2462–73. doi: 10.1038/s41401-022-00887-6 35288674 PMC9525650

[46] Chang S Tang M Zhang B Xiang D Li F . Ferroptosis in inflammatory arthritis: A promising future. Front Immunol. (2022) 13:955069. doi: 10.3389/fimmu.2022.955069 35958605 PMC9361863

[47] Xie Y Hou W Song X Yu Y Huang J Sun X . Ferroptosis: process and function. Cell Death Diff. (2016) 23:369–79. doi: 10.1038/cdd.2015.158 26794443 PMC5072448

[48] Wallace HL Wang L Gardner CL Corkum CP Grant MD Hirasawa K . Crosstalk between pyroptosis and apoptosis in hepatitis C virus-induced cell death. Front Immunol. (2022) 13:788138. doi: 10.3389/fimmu.2022.788138 35237259 PMC8882739

[49] Novack DV Mbalaviele G . Osteoclasts-key players in skeletal health and disease. Microbiol Spectr. (2016) 4:MCHD-0011-2015. doi: 10.1128/microbiolspec.MCHD-0011-2015 27337470 PMC4920143

[50] Yang J Dong D Luo X Zhou J Shang P Zhang H . Iron overload-induced osteocyte apoptosis stimulates osteoclast differentiation through increasing osteocytic RANKL production *in vitro*. Calcif Tissue Int. (2020) 107:499–509. doi: 10.1007/s00223-020-00735-x 32995951

[51] Ishii K Fumoto T Iwai K Takeshita S Ito M Shimohata N . Coordination of PGC-1beta and iron uptake in mitochondrial biogenesis and osteoclast activation. Nat Med. (2009) 15:259–66. doi: 10.1038/nm.1910 19252502

[52] Tanaka Y Nakayamada S Okada Y . Osteoblasts and osteoclasts in bone remodeling and inflammation. Curr Drug Targ-Inflamm Allergy. (2005) 4:325–8. doi: 10.2174/1568010054022015 16101541

