# Supplementary figures and images for: Iron overload disrupts bone homeostasis via TfR1-dependent ferroptosis and cGAS/STING-driven pyroptosis in pyogenic spondylitis
Source: Front Immunol. 2026 Jun 30;17:1760434. doi: 10.3389/fimmu.2026.1760434 (PMC13364566; doi:10.3389/fimmu.2026.1760434)

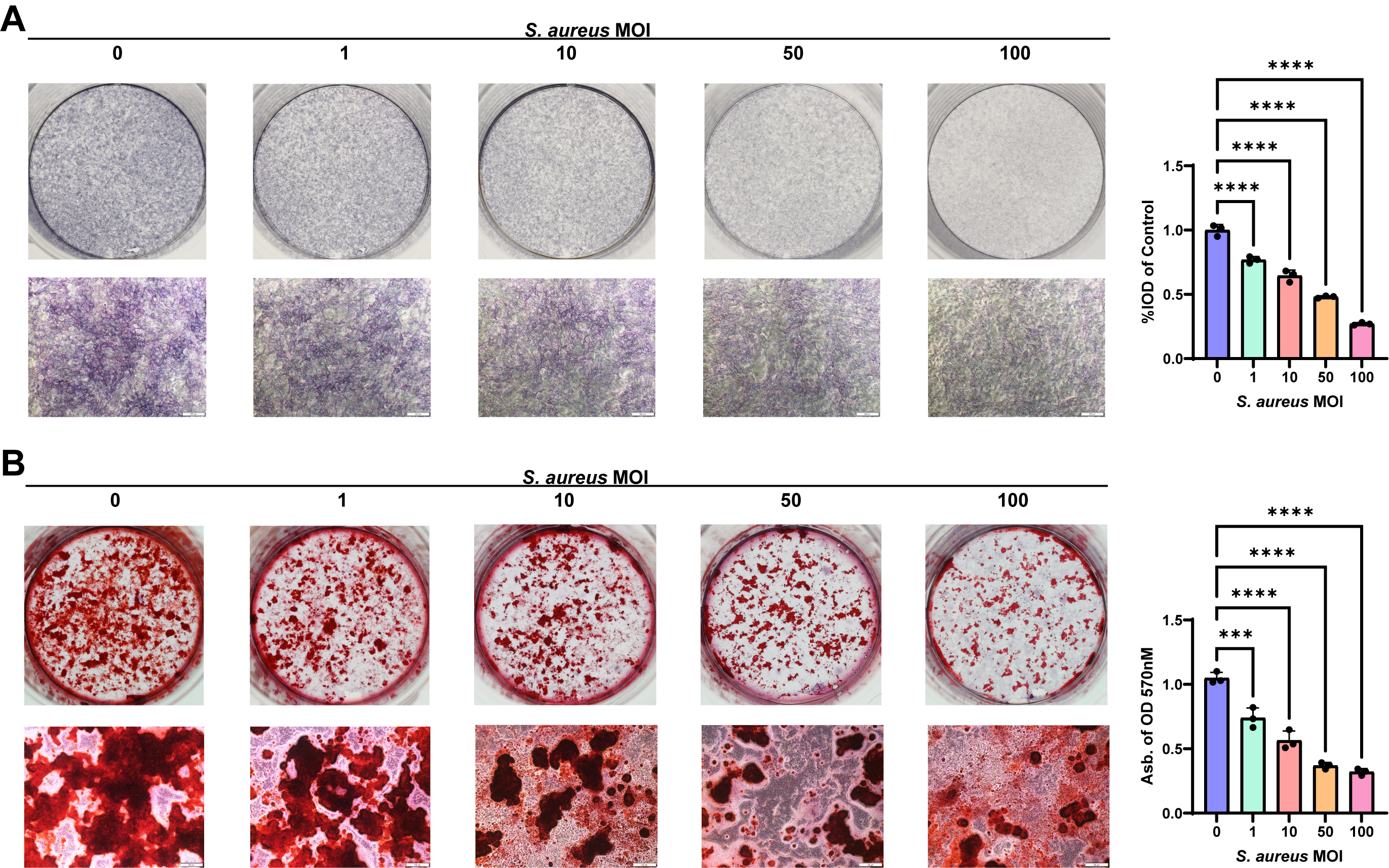

Supplement: Supplementary Figure 1 — S. aureus inhibited MC3T3-E1 cells osteogenic differentiation through inducing ferroptosis. (A) ALP staining for MC3T3-E1 cells under different MOI. (Scale bar =200μm). Semi-quantitative analysis indicated the damaged osteogenesis. (B) Alizarin Red staining for calcium deposition and its semi-quantitative analysis under diverse MOI in MC3T3-E1 cells (Scale bar =100μm). Data are presented as mean ± SD, n = 3. ***p < 0.001, and ****p < 0.0001. [file Image1.tif]

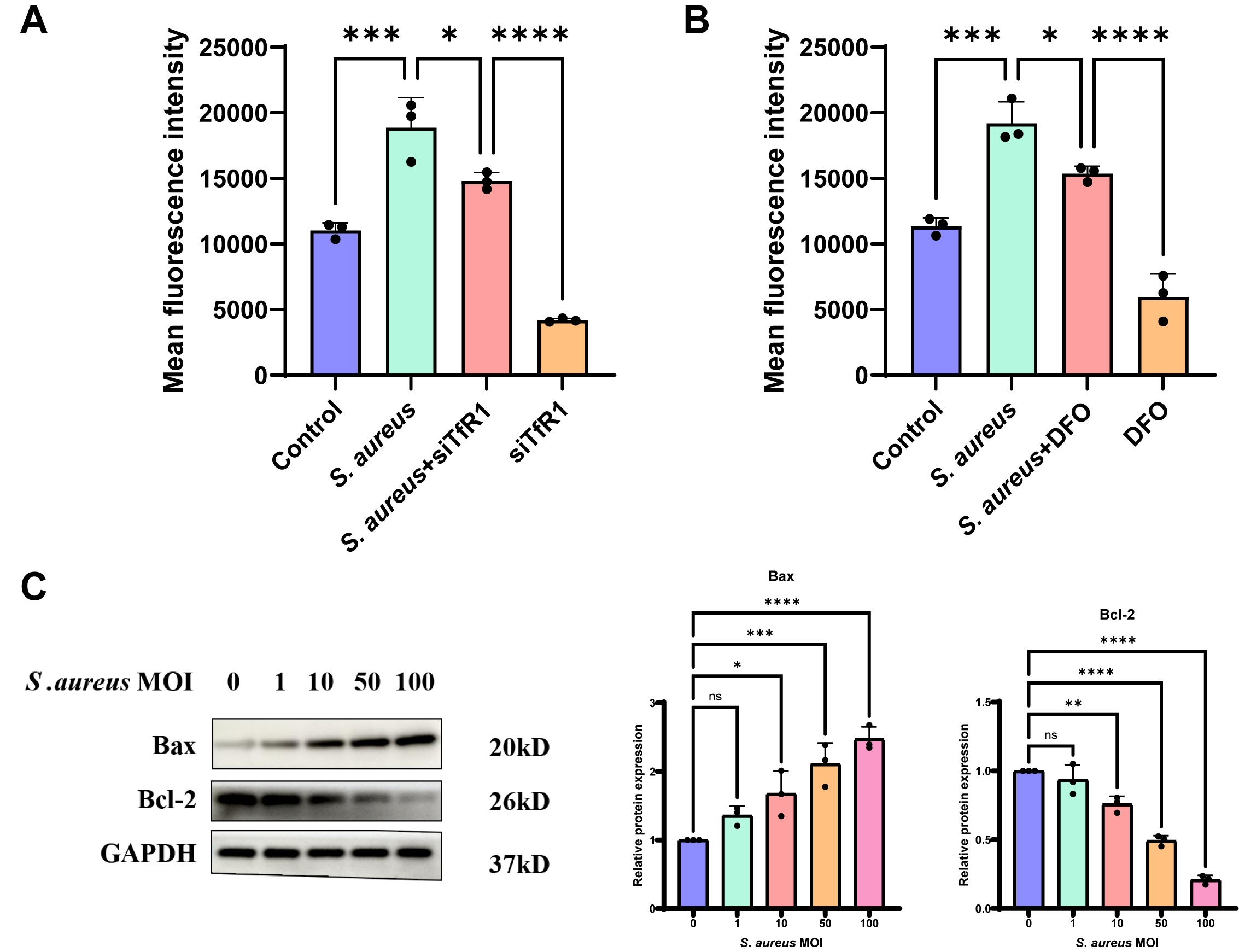

Supplement: Supplementary Figure 2 — Inhibition of TfR1 alleviated S. aureus induced mitochondrial dysfunction and apoptosis. (A, B) Flow cytometric analysis was conducted to quantify the ROS production. (C) Western blot for Bax and Bcl-2 protein under different MOI. Data are presented as mean ± SD, n = 3. ns, p > 0.05, *p < 0.05, **p < 0.01, ***p < 0.001, and ****p < 0.0001. [file Image2.tif]
